# Atypical Teratoid/Rhabdoid Tumor of the Posterior Fossa Mimicking Medulloblastoma in an Infant: A Case Report

**DOI:** 10.7759/cureus.103516

**Published:** 2026-02-13

**Authors:** Abdelali Yahia, Adlene Saadna

**Affiliations:** 1 Neurosurgery, Ouargla Regional University Hospital, Ouargla, DZA; 2 Pathology, Bechar Regional University Hospital, Bechar, DZA

**Keywords:** atypical teratoid/rhabdoid tumor, central nervous system, embryonal tumors, ini1 loss, medulloblastoma, pediatric brain tumors, posterior fossa tumors

## Abstract

Atypical teratoid/rhabdoid tumor (AT/RT) is a rare and highly aggressive central nervous system (CNS) neoplasm that predominantly affects children under three years of age. In the posterior fossa, AT/RT frequently mimics medulloblastoma (MB) on both radiological and histopathological evaluation. Owing to significant morphological overlap, a definitive diagnosis cannot be established on histology alone and requires immunohistochemical (IHC) confirmation, particularly demonstration of loss of nuclear integrase interactor 1 (INI1) expression.

We report the case of a seven-month-old male infant who presented with signs of intracranial hypertension and rapidly progressive symptoms attributable to posterior fossa mass effect. Neuroimaging revealed a posterior fossa mass causing marked compression of the fourth ventricle and brainstem, resulting in obstructive hydrocephalus. Magnetic resonance imaging demonstrated heterogeneous contrast enhancement, initially suggestive of MB. Cerebrospinal fluid diversion was performed, followed by tumor biopsy and partial surgical resection. Although intraoperative findings and initial histopathological examination were suggestive of MB, subsequent IHC analysis demonstrated complete loss of nuclear INI1 expression, confirming the diagnosis of AT/RT. The patient subsequently received adjuvant chemotherapy.

AT/RT should be strongly considered in infants presenting with posterior fossa tumors, particularly when radiological and histopathological features resemble MB. Early tissue diagnosis and routine use of INI1/SMARCB1 IHC are essential for accurate differentiation from morphologically similar entities, ensuring appropriate therapeutic management and prognostic assessment.

## Introduction

Medulloblastoma (MB) is the most common malignant posterior fossa tumor in children, accounting for approximately 15-20% of all pediatric brain tumors and 30-40% of posterior fossa neoplasms [1-5]. In early childhood, the principal malignant differential diagnosis of MB in the posterior fossa is atypical teratoid/rhabdoid tumor (AT/RT) [6]. AT/RT was first recognized as a distinct clinicopathological entity by Rorke et al. in 1993 [7]. Although rare overall, AT/RT accounts for approximately 1-2% of pediatric central nervous system (CNS) tumors [3,8-10] and is the most common malignant CNS tumor in children younger than three years of age [9,11]. In infants under one year of age, AT/RT accounts for up to 40-50% of CNS malignancies [11].

AT/RT predominantly arises in the cerebellum in young children and is characterized by an aggressive clinical course and poor prognosis [12]. Owing to substantial overlap in clinical presentation, radiological characteristics, and histopathological features, AT/RT is frequently misdiagnosed as MB. Reliable differentiation between these entities cannot be achieved based on morphology alone and requires immunohistochemical (IHC) and molecular analyses. AT/RT is characteristically associated with deletions of chromosome 22q and inactivation of the INI1/SMARCB1 (hSNF5) gene, with loss of nuclear INI1/SMARCB1 expression considered a diagnostic hallmark [13,14].

In this report, we present a rare case of posterior fossa AT/RT in a seven-month-old infant that was initially misdiagnosed as MB, highlighting the diagnostic challenges posed by this highly aggressive tumor and emphasizing the importance of early IHC confirmation.

## Case presentation

A seven-month-old male infant was brought to the emergency department with a four-week history of nonspecific symptoms, including progressive lethargy and recurrent vomiting. According to his parents, the patient had also developed decreased appetite and weight loss during this period. There was no personal or family history of malignancy, including CNS tumors. The pregnancy was uneventful, with no reported maternal tobacco use, illicit drug exposure, or other relevant risk factors.

On physical examination, the patient was alert but irritable. No focal neurological deficits were identified. Motor and sensory examinations were normal, and pupillary reflexes and extraocular movements were intact.

**Figure 1:**
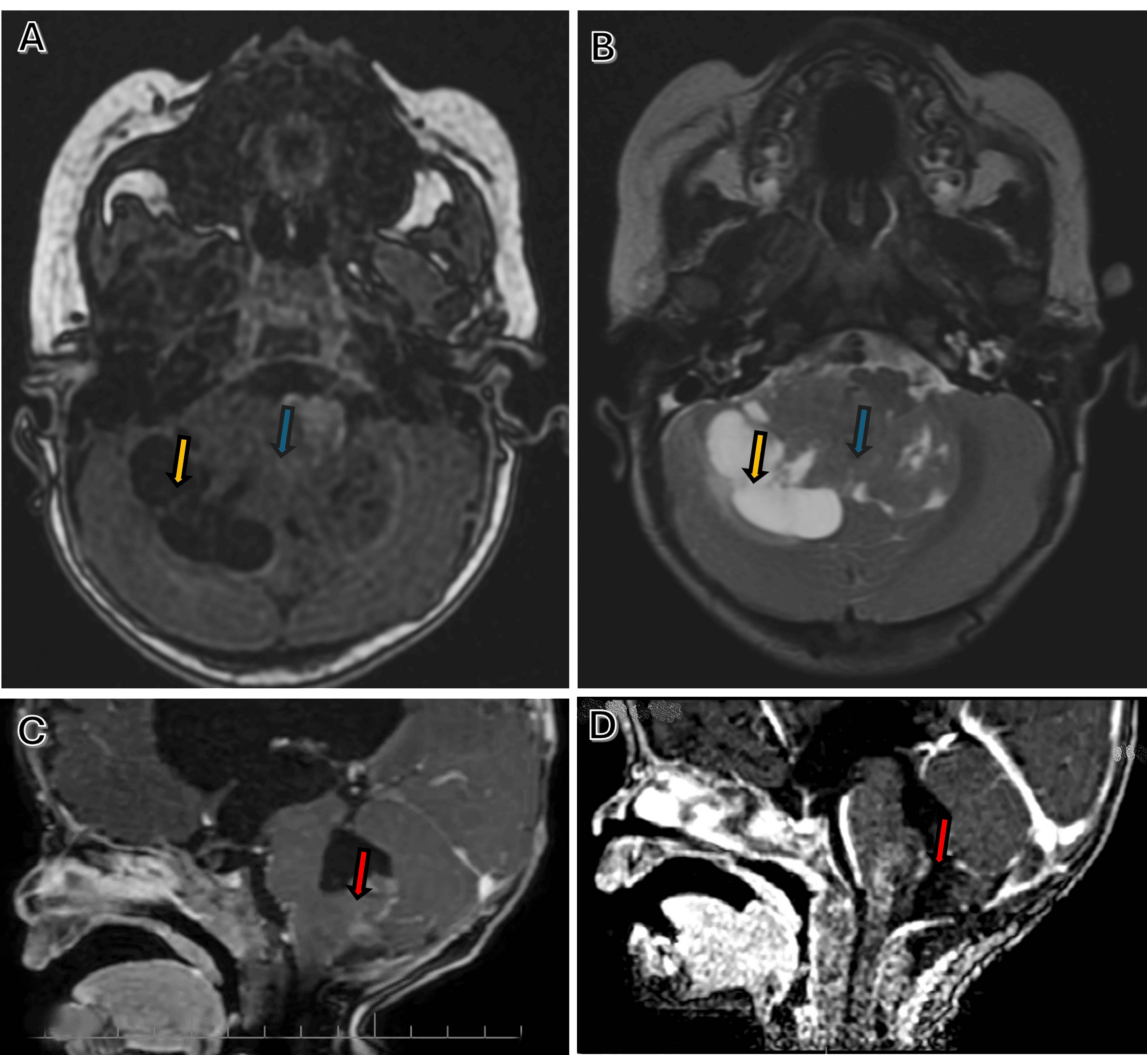
MRI of the posterior fossa tumor Axial T1-weighted (A) and T2-weighted (B) MRI sequences demonstrate a heterogeneous posterior fossa mass with mixed solid (blue arrow) and cystic (yellow arrow) components, arising from the cerebellum and extending into the fourth ventricle with extension toward the CPA. Sagittal contrast-enhanced T1-weighted images show the preoperative appearance of the tumor (red arrow) (C) and the immediate postoperative status on postoperative day 1 (red arrow) (D), confirming partial tumor resection. MRI: magnetic resonance imaging, CPA: cerebellopontine angle

Initial magnetic resonance imaging (MRI) revealed a heterogeneously contrast-enhancing posterior fossa mass measuring approximately 3.1 × 3.6 × 4.8 cm, with mixed solid and cystic components. The lesion appeared to arise from the cerebellum and extended into the fourth ventricle with extension toward the cerebellopontine angle (CPA) (Figure 1). Marked obstructive hydrocephalus with associated periventricular signal changes was present. Although imaging demonstrated features commonly seen in embryonal posterior fossa tumors, the radiological appearance was most suggestive of MB. Given the substantial overlap in imaging characteristics between MB and AT/RT in this age group, radiologic differentiation was not possible, and an initial working diagnosis of MB was made.

To address the obstructive hydrocephalus, a ventriculoperitoneal (VP) shunt was placed, followed by a suboccipital craniotomy using a telovelar tonsillar approach. Intraoperatively, the tumor was soft in consistency and appeared to originate from the cerebellar tonsils, extending into the fourth ventricle and closely abutting the caudal aspect of the brainstem. Given the lesion's infiltrative nature, partial resection with biopsy was performed, and representative tissue was submitted for histopathological evaluation.

The postoperative course was uneventful, and the patient remained neurologically stable without complications. Postoperative MRI confirmed partial tumor resection (Figure 1).

**Figure 2:**
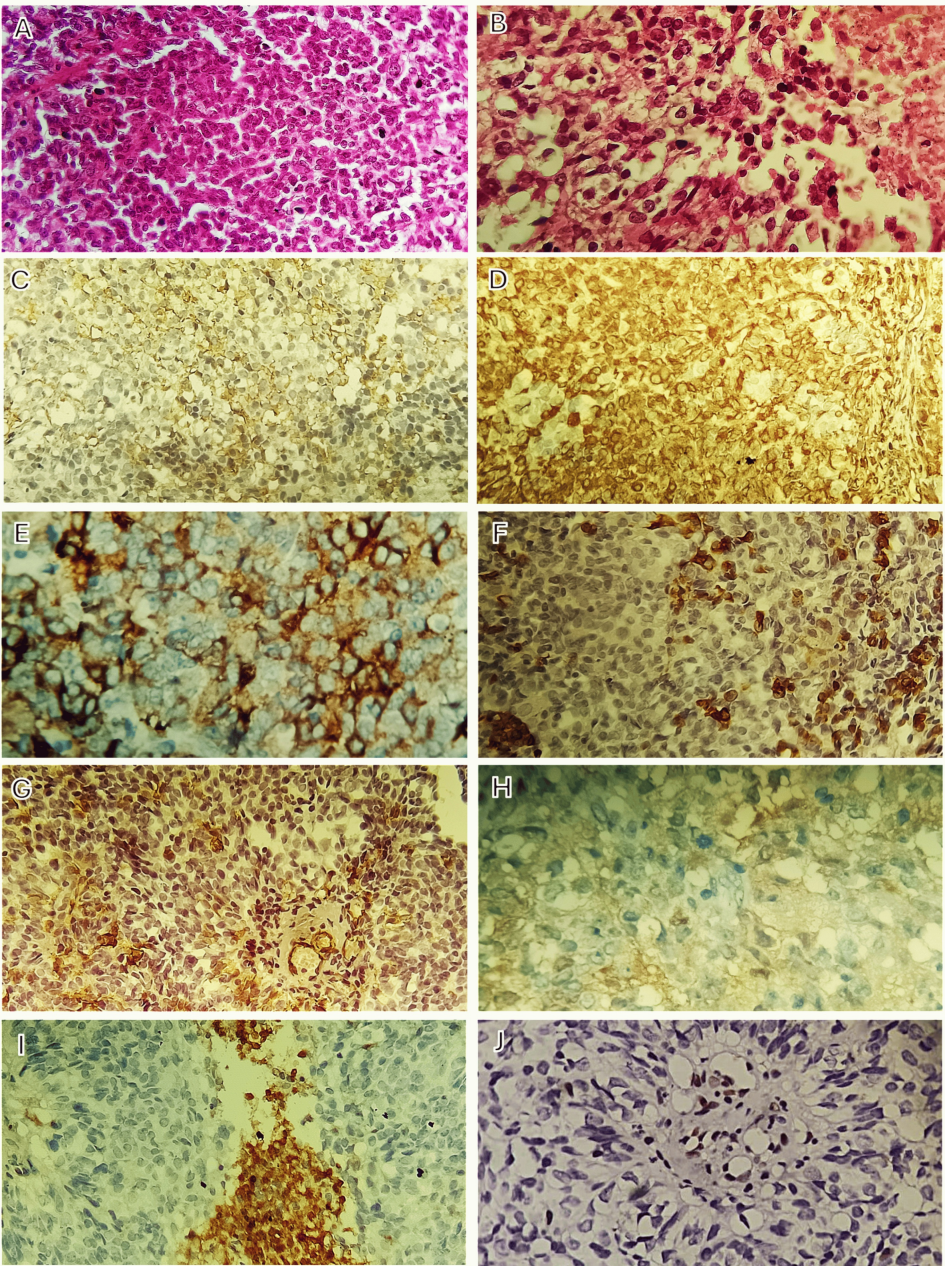
Histopathological and IHC findings Histopathological examination reveals a highly cellular tumor composed of small round blue cells with rhabdoid morphology, brisk mitotic activity, and areas of necrosis (H&E: A, B). IHC analysis demonstrates tumor cell positivity for EMA (C), vimentin (D), and synaptophysin (E), with focal positivity for GFAP (F) and SMA (G). Tumor cells are negative for chromogranin (H) and CD45 (I). Complete loss of INI1/SMARCB1 expression is observed in tumor cells, whereas lymphocytes and endothelial cells retain nuclear staining, which serve as internal positive controls (J) and are diagnostic of AT/RT. H&E: hematoxylin and eosin, EMA: epithelial membrane antigen, GFAP: glial fibrillary acidic protein, SMA: smooth muscle actin, INI1/SMARCB1: nuclear integrase interactor 1, IHC: immunohistochemical

Histopathological examination revealed a highly cellular tumor composed of small round blue cells with rhabdoid morphology, brisk mitotic activity, and areas of necrosis. IHC analysis demonstrated tumor cell positivity for epithelial membrane antigen (EMA), vimentin, and synaptophysin, with focal positivity for glial fibrillary acidic protein (GFAP) and smooth muscle actin (SMA). Tumor cells were negative for CD45 and chromogranin. Complete loss of nuclear integrase interactor 1 (INI1/SMARCB1) protein expression was observed in tumor cells, whereas retained nuclear staining in non-neoplastic cells served as an internal positive control (Figure 2). These findings were diagnostic of AT/RT, World Health Organization grade 4.

Following discharge, the patient was referred to a tertiary oncology center, where he received intensive, protocol-based multiagent chemotherapy. Despite treatment, the disease progressed, and the patient died five months after surgery.

## Discussion

AT/RT is one of the most aggressive malignant tumors of the CNS. Although it accounts for approximately 1-2% of all pediatric brain tumors, it represents up to 10-20% of malignant CNS neoplasms in children younger than three years of age [13,14]. Rare cases have also been reported in adults [15]. Despite advances in multimodal treatment strategies, including surgery, chemotherapy, and radiotherapy, the prognosis of AT/RT remains significantly poorer than that of MB [16]. Moreover, AT/RT typically presents at a much younger age than MB or other embryonal tumors [17].

The clinical presentation is nonspecific and largely determined by tumor location [17,18]. In most cases, symptoms are related to increased intracranial pressure secondary to obstructive hydrocephalus, as observed in the present case. The absence of distinctive clinical features frequently contributes to delayed diagnosis or initial misclassification as more common pediatric posterior fossa tumors [17].

Radiological differentiation of AT/RT from other posterior fossa neoplasms remains challenging. Although several imaging characteristics have been described, AT/RT typically demonstrates heterogeneous contrast enhancement and may contain cystic, necrotic, or hemorrhagic components [6,19,20]. However, these features lack sufficient specificity to distinguish AT/RT from MB reliably. In infants and young children, posterior fossa tumors are most commonly presumed to be MB, rendering initial misdiagnosis understandable. Furthermore, because many AT/RTs arise in the posterior fossa and may contain primitive neuroectodermal elements, they are frequently mistaken for MB or other embryonal tumors. In this context, IHC demonstration of loss of INI1/SMARCB1 expression remains the most reliable diagnostic criterion [14].

Given the findings in the present case and the apparent scarcity of reported AT/RT cases, this entity may remain underrecognized. AT/RT should therefore be strongly considered in children younger than three years presenting with infratentorial tumors, particularly those that are eccentric, extend toward the CPA, and radiologically mimic MB [6]. Routine assessment of INI1/SMARCB1 expression is essential for accurate diagnosis and for distinguishing AT/RT from MB and other embryonal tumors, thereby facilitating appropriate therapeutic management and prognostic counseling [14].

## Conclusions

AT/RT is a rare but highly aggressive CNS neoplasm that predominantly affects infants and young children and poses a significant diagnostic challenge in the posterior fossa. As demonstrated in this case, AT/RT may closely mimic MB in clinical presentation, neuroimaging, and even initial histopathological assessment, leading to potential misdiagnosis. Reliance on morphology alone is insufficient; definitive diagnosis requires IHC confirmation, particularly the demonstration of loss of nuclear INI1/SMARCB1 expression, which remains the defining diagnostic hallmark.

Early and accurate identification of AT/RT is critical, as prognosis, therapeutic strategies, and counseling differ substantially from those of other embryonal posterior fossa tumors. This case underscores the importance of maintaining a high index of suspicion for AT/RT in infants presenting with posterior fossa masses. Routine incorporation of INI1/SMARCB1 immunohistochemistry into diagnostic workflows is essential to ensure accurate classification, guide appropriate multidisciplinary management, and provide realistic prognostic information to families.
